# Platelet Activating Factor Blocks Interkinetic Nuclear Migration in Retinal Progenitors through an Arrest of the Cell Cycle at the S/G2 Transition

**DOI:** 10.1371/journal.pone.0016058

**Published:** 2011-01-27

**Authors:** Lucianne Fragel-Madeira, Tamara Meletti, Rafael M. Mariante, Robson Q. Monteiro, Marcelo Einicker-Lamas, Robson R. Bernardo, Angela H. Lopes, Rafael Linden

**Affiliations:** 1 Institute of Biophysics, Universidade Federal do Rio de Janeiro, Rio de Janeiro, Brazil; 2 Institute of Medical Biochemistry, Universidade Federal do Rio de Janeiro, Rio de Janeiro, Brazil; 3 Institute of Chemistry, Universidade Federal do Rio de Janeiro, Rio de Janeiro, Brazil; 4 Institute of Microbiology, Universidade Federal do Rio de Janeiro, Rio de Janeiro, Brazil; Center for Regenerative Therapies Dresden, Germany

## Abstract

Nuclear migration is regulated by the LIS1 protein, which is the regulatory subunit of platelet activating factor (PAF) acetyl-hydrolase, an enzyme complex that inactivates the lipid mediator PAF. Among other functions, PAF modulates cell proliferation, but its effects upon mechanisms of the cell cycle are unknown. Here we show that PAF inhibited interkinetic nuclear migration (IKNM) in retinal proliferating progenitors. The lipid did not, however, affect the velocity of nuclear migration in cells that escaped IKNM blockade. The effect depended on the PAF receptor, Erk and p38 pathways and Chk1. PAF induced no cell death, nor a reduction in nucleotide incorporation, which rules out an intra-S checkpoint. Notwithstanding, the expected increase in cyclin B1 content during G2-phase was prevented in the proliferating cells. We conclude that PAF blocks interkinetic nuclear migration in retinal progenitor cells through an unusual arrest of the cell cycle at the transition from S to G2 phases. These data suggest the operation, in the developing retina, of a checkpoint that monitors the transition from S to G2 phases of the cell cycle.

## Introduction

Progression of the cell cycle is precisely controlled by checkpoints, the signaling networks that allow cells to monitor successive events and ensure ordered cell proliferation and genomic stability. These include a mechanism that prevents transition from G1 to S phase while the DNA replication machinery is not ready; an intra-S checkpoint, which monitors the progress of DNA synthesis; surveillance over DNA damage throughout the cell cycle; and a mechanism in M-phase, which ensures that completion of cell division awaits proper alignment and attachment of all chromosomes to the mitotic spindle ([1], [2], [3], [4] for reviews). More importantly, checkpoints are not only induced by occasional danger signals, but operate continuously during the unperturbed cell cycle to guarantee smooth and safe phase transitions [5], [6].

Phases of the cell cycle in the nervous system, including both the early neuroepithelium and ventricular zone of the mammalian cerebral hemispheres, as well as the corresponding neuroblastic layer (NBL) of the developing vertebrate retina, uniquely correlate with the to-and-fro movement of the nuclei of proliferating cells, known as interkinetic nuclear migration (IKNM). In each cell division cycle, the nucleus of an elongated neural precursor that enters G1 migrates from the apical end towards the basal side, wherein DNA is duplicated. Upon completion of S phase, the nucleus migrates back towards the lumen of the neural tube, reaching the apical end of the cell, where mitosis takes place [7], [8].

Current data support the hypothesis that IKNM is regulated by unevenly distributed signals associated with the distinct phases of the cell cycle [9]. An example is asymmetric Notch signaling, which results from asymmetric mRNA stability in distinct phases of the cell cycle, and controls neurogenic exit from the cell cycle by imposing upon the basal to apical nuclear migration in retinal progenitor cells [10], [11].

Studies of the mechanisms that control the movement of the nuclei proper have implicated microtubules [12], [13], dynein and centrosome proteins [14], [15], SUN1/2 and Syne/Nesprin-1/2 complexes [16], as well as actomyosin-dependent forces based on myosin II in nuclear transport along the IKNM path [17], [18], [19]. Recent work showed also that interkinetic nuclear movement involves Ca^2+^ transients dependent on functional gap junction hemichannels [20], [21]. The interplay among the involved molecules and the mechanical events is, nonetheless, still debatable.

Coordinated cell and nuclear movements are critical events of embryonic development [22], and developmentally regulated migration of newly-generated neurons within the mammalian brain enrolls evolutionarily conserved mechanisms similar to the long range nuclear migration seen in the growing hyphae of filamentous fungi. For example, striking similarities were reported between the sequences of the proteins NUDF of *Aspergillus nidulans* and LIS1 of mammals, as well as in their mechanisms of action upon the dynein/dynactin components of the cytoskeleton [23], [24]. Indeed, mutations in NUDF block migration of nuclei in growing hyphae [25], whereas mutations in LIS1 deregulate IKNM [14], [26].

LIS1 dimers constitute the regulatory subunit of the enzyme platelet activating factor acetyl-hydrolase (PAFAH), which metabolizes and inactivates the pleiotropic lipid messenger platelet activating factor (PAF, 1-*O*-alkyl-2-*O*-acetyl-*sn*-glycero-3-phosphorylcholine) [27], [28]. Engagement of a G protein-coupled membrane receptor for PAF (PAFR) leads to activation of phospholipases A_2_, C and D, of the PI3-K and MAPK pathways, such as Erk and p38, and inhibition of adenylyl cyclase in various cells and tissues [29]. In addition, almost all signaling pathways induced by PAFR are networked with tyrosine kinases [30].

Notwithstanding somewhat conflicting results, both the presence of PAF, as well as the hydrolytic activity of PAFAH affect neuronal migration both *in vivo* and *in vitro* [31], [32], [33], [34]. Based on both the functional similarities between LIS1 and NUDF, and on the known effects of PAF upon neuronal migration, we tested the effects of PAF upon IKNM in the developing rat retina. We found that a PAF-like lipid is produced within the developing retina, and that PAF affects interkinetic nuclear migration. Unexpectedly, however, the blockade of IKNM was not due to interfering with nuclear movement, but rather unusually to the induction of a Chk1-dependent cell cycle arrest at the S/G2 transition, in the absence of either DNA synthesis blockade or damage.

## Methods

All procedures complied with the Guidelines for the Care and Use of Laboratory Animals, from the ARVO Statement for the Use of Animals in Ophthalmic and Vision Research, and were approved by the Committee for the Use of Experimental Animals of the Institute of Biophysics.

### Materials

PAF, C-PAF, lyso-PAF, PD98059 and LY294002 were from Calbiochem; WEB2086 was a gift from Boehringer Ingelheim; SB218078 was from Glaxo-Smith Kline; Forskolin, cheleritrin chloride, SB239063, caffeine, 5-Bromo-2-deoxyuridine (BrdU), DNAse (D4513), Hepes and glutamine from Sigma; Basal Medium of Eagle, gentamicin from Gibco BRL; antibody against BrdU (RPN 202) from Amersham Pharmacia, antibody against phospho-histone H3 (9701) from Cell Signaling; protein A/G plus agarose and antibodies against PAF receptor (sc-8744) and CHK1 were from Santa Cruz Biotechnology; and antibody against phospho-histone H2AX Ser 139 (clone JBW301) from Upstate Biotechnology. HRP-ABC kit was from Vector, fluorescent secondary antibodies were from Molecular Probes and Jackson ImmunoResearch.

### Nuclear labeling and tissue culture

Lister hooded rats at 2 days postnatal were anaesthetized by hypothermia and a solution of BrdU diluted in distilled water plus 0.007N of NaOH was injected intraperitoneally (60 mg/kg b.w.). The animals were killed instantaneously by decapitation at 30 min after the injection, their eyes were quickly removed and retinal explants of about 1 mm×1 mm, were prepared as described [35]. In each experiment, tissue fragments from all retinae were pooled and mixed before explants corresponding to approximately 2 retina per data point were distributed among the experimental groups. The explants were placed in 2 cm^2^ plates containing 500 µL of BME with 2 mM glutamine and 10 µg/mL gentamicin. The dissection procedure took less than 30 min. Culture plates were kept at 37°C in the presence of various reagents, for 3 hours except where noted. This culture period allows BrdU-labeled cells that leave S phase, to proceed along G2 and M phases of the cell cycle without re-entering G1 of the following cycle [36].

### Histology

Tissue was fixed with 4% paraformaldehyde in sodium phosphate buffer pH 7.4 (4% PF), and then oriented for transverse sections under a dissecting microscope in an aluminum chamber filled with OCT embedding medium. Transverse sections were cut in a cryostat. Sections of the retina *in situ* were cut frozen from eyes fixed with 4% PF, after infiltration of sucrose, and were mounted on 200 µg/mL poly-L-lysine coated glass slides.

For immunolabeling of single cells, the explants were enzymatically dissociated with 0.125% trypsin in a calcium- and magnesium-free (CMF) saline for 10 min at 37°C and treated with 0.2 mg/mL DNAse I for 5 min. The cells were allowed to adhere in a slide covered with 200 µg/mL poli L-lysine for 30 min at 37°C, and then fixed with 4%PF for 30 min.

### Immunohistochemistry

Sections of retinal explants were incubated with 0.5% Triton X-100 in phosphate buffered-saline (PBS) pH 7.4, for 15 min, washed and incubated with 1% BSA in PBS for 30 min, followed by primary antibody either for 1 hour at 37°C or overnight at room temperature. Then, the sections were washed, incubated with secondary antibodies, and stained with an HRP-ABC kit using DAB as chromogen. In the cases where both antibodies to phospho-histone H3 and BrdU were used, the slides were heated in 10 mM citrate buffer at pH 6.0 in a microwave oven for antigen retrieval [37], and developed with Alexa 555- and 488-conjugated fluorescent secondary antibodies, respectively.

### Immunocytochemistry for phospho-histone H2A.X

Following antigen retrieval, dissociated cells from either control or treated explants were incubated with a mouse monoclonal antibody against phospho-Histone H2A.X for 2 hours at room temperature and developed with Alexa 555-conjugated anti-mouse antibody. SYTOX® Green nucleic acid stain was used to visualize retinal cell nuclei. Counts of γ-H2AX foci were done at 630X magnification for at least three hundred cells, under indirect fluorescence in a Zeiss Axiophot microscope.

### Tritiated thymidine assay

To measure DNA synthesis *in vitro*, retinal explants were incubated with [^3^H]-Thymidine, either during the 3-hour experimental period or only during the last hour, washed and homogenized following overnight incubation with 0.4 M NaOH. Aliquots of the homogenates were precipitated with 100 volumes of 10% trichloroacetic acid, and collected on GF/A Whatman filters. Following additional washes, the filters were oven-dried at 110°C, and counted in a Packard model 1600TR liquid scintillation analyzer.

### Detection and counting of migrating nuclei

In the P2 rat retinae an S-phase zone corresponds to between one-half and two-thirds of the transversal length of the neuroblastic layer ([38] and unpublished results), and other studies have shown that movements of the nuclei of neural progenitor cells are minimal during the whole period of DNA duplication in S-phase [39]. Therefore, BrdU-labeled nuclei within the apical (outer) third of the NBL were scored as migrating. Counts were made of those, as well as of all labeled nuclei within rectangular fields of 122 µM width across the whole extent of the NBL, at 1000X magnification under differential interference contrast in a Zeiss Axiophot microscope. For each data point, three fields were examined from each of three distinct explants. A *nuclear migration index* was defined as the fraction of nuclei within the outer third of the NBL, and a *BrdU incorporation index* was defined as the total count of labeled nuclei within the counting field (Figure S1).

### Velocity of nuclear movement

To estimate the velocity of nuclear movement, retinae from 2 day-old rats were dissected and explants were cut radially to allow examination of nuclear migration along the full central-peripheral gradient of retinal differentiation. In this particular experiment, 3 µg/mL BrdU were added *after* explantation for 30 minutes, then the medium was changed and the explants were either maintained in control medium or treated with 0.3 nM PAF for 0.5, 1, 2, or 3 hours.

Following the same procedures as above, the immunolabeled explant sections were photographed at high resolution under differential interference contrast, using a Zeiss Axiocam digital camera on an Axiophot microscope, driven by Axiovision software. In each photomontage of the whole section, 5 evenly spaced lines were drawn perpendicular to the retinal surface, each extending from the basal (inner) to the apical (outer) border of the NBL, at one-sixth to five-sixths of the total eccentricity from the central border of the section. Then, the positions of the 2 nuclei located within 50 µm distance of the marked line that had moved the furthest towards the apical border, were measured, averaged and expressed as percentage of the total depth of the NBL (Figure S2). The results were pooled within each of 10 evenly spaced intervals, from the basal to the apical border of the NBL, of which 10% correspond to the innermost, while 100% correspond to the outermost (mitotic) stratum. Data were collected for either control or PAF-treated explants at 4 time points from 2 independent experiments, each from 3–4 explants in duplicate. This procedure allowed us to map a wavefront of IKNM from extensive sampling at various intervals of time after BrdU labeling of the S-phase nuclei.

### Purification and identification of retinal lipids

Phospholipids from the neuroretina were extracted and partially purified by bi-dimensional thin layer chromatography (bi-TLC), following published procedures [40]. The phospholipids were detected with iodine vapor, and the spot corresponding to a commercial standard of PAF was scraped from the plate [41]. HPLC analysis was done using a Varian Chromatography Star System connected to a pump (model Dynamax SD-200) with a *loop* of 20 µL. The partially purified fraction, obtained from bi-TLC, was separated in a reverse-phase column (LiChrosphere® 60 RP-18, 12,5 cm×4 mm I.D. 5 µm, Merck), and eluted in an isocratic mobile phase of methanol:H_2_O (20∶80 v/v) at a flow rate of 0,7 ml/min. Each sample was detected by UV absorbance at 205 nm. Commercial PAF, as well as other pure lipids were similarly treated to serve as controls.

### Purified Muller glial cell culture

Muller cell cultures were prepared from 2-day-old Lister hooded rats, following a published procedure [42]. Briefly, mechanically dissociated retinal cells were plated onto polyornithine-coated 25 cm^2^ polystyrene flasks, in DMEM/F12 medium supplemented with 10% fetal bovine serum. The cultures were incubated for 20–30 days until confluence, at the time of which nearly all neurons had degenerated, leaving a highly purified culture of Muller glial cells. The phospholipids were extracted as described above, and compared with extracts from neural retina, the vitread vascular layer, or the back of the eye containing the sclera, choroid and immature pigment epithelium, mechanically dissected from the eyes.

### Platelet aggregation assay

The partially purified fraction obtained from bi-TLC was tested also in a platelet aggregation assay, following published procedures [43]. Rabbit blood platelets (3 to 4×10^5^ cells/mL) were treated with 1 mM calcium and 5 µL of agonists, in Tyrode's buffer. The assay was analyzed in an aggregometer, with monitoring time of 5 min.

### Western Blot

Total protein extracted from the retinae of 2 day-old rats were separated by 10% SDS-PAGE, transferred onto nitrocellulose membrane, and probed with a polyclonal goat anti-PAF receptor antibody, followed by horseradish peroxidase-conjugated anti-goat secondary antibody, and visualized using a chemiluminescent ECL kit (GE Healthcare).

### Measurement of Chk1 activity

Chk1 kinase assay was done *in vitro* using GST-Cdc25C_200–256_ as substrate as previously described [44]. Briefly, plasmid pGEX-KG-GST-Cdc25C_200–256_ was introduced into cells of the transformation competent BL21Star *E. coli* strain by electroporation. GST-Cdc25C_200–256_ fusion protein was affinity-purified using a glutathione sepharose 4B matrix (GS4B – Amersham Pharmacia) following a batch method described by the manufacturers. After incubation with 0.3 nM PAF for 45 min at 37°C, Chk1 was immunoprecipitated from 1 mg of total retinal proteins by incubation with 20 µg of a Chk1 specific antibody and 40 µl of A/G protein-agarose overnight at 4°C. For *in vitro* kinase assay, the precipitate was incubated with incomplete kinase buffer (50 mM Tris-HCl pH 7.4, 1 mM DTT, 10 mM MgCl_2_) in the presence of 10 µM ATP, 10 µCi γ-ATP^32^ and 5 ng of purified Cdc25C_200–256_ for 25 minutes at 30°C. The samples were separated by SDS-PAGE and transferred to a nitrocellulose membrane, which was exposed to a phosphor screen using a phosphor cassette overnight at room temperature and developed by phosphorimager scanning with a Storm 860 (GE). The same nitrocellulose membrane was probed with an antibody to Chk1 as a control and results were analyzed using Image J 1.42q software (NIH-USA).

### Flow cytometry

Flow cytometric analyses were conducted on a FACScalibur cytometer (BD Biosciences). Retinal explants, prepared as described previously, were enzymatically dissociated with 0.125% trypsin in CMF solution for 10 min at 37°C and treated with 0.2 mg/mL DNAse I for 5 min. Dissociated cells were fixed with 4% PF for 30 min, washed, subject to antigen retrieval and incubated with a rabbit polyclonal antibody against cyclin B1 at a pre-titrated dilution for 2 h at 37°C and further developed with DyLight 488-conjugated anti-rabbit. Then, a mouse monoclonal antibody against BrdU was added for 1 hour at room temperature, and developed with DyLight 649-conjugated anti-mouse. Data acquisition from at least 10,000 cells was carried out with CellQuest software (BD Biosciences), and the Summit MoFlo software (Cytomation) was used for further analyses. Cyclin B1 fluorescence intensity was measured among the BrdU positive cell population, as the difference between the median values of cells labeled with the anti-cyclin B1 antibody and those stained with secondary antibody alone, and expressed as percentage of control.

### Statistical analysis

All quantitative data are expressed means ± standard errors of the mean. Statistical analysis was done using Prism v.5.0, by analysis of variance followed by intergroup planned comparisons with the Tuckey test. Statistical significance was set at p<0.05.

## Results

### Both PAF and its receptor are expressed in the developing rat retina

Both uni- and bi-dimensional thin layer chromatograms of lipid extracts of the neonatal rat retina showed a distinct spot coincident with commercial PAF subject to the same conditions (Figures S3A, S4A). This partially purified fraction, scraped and eluted from the silica plate of bi-dimensional chromatograms, is here referred to as PAF-*like*.

**Figure 1:**
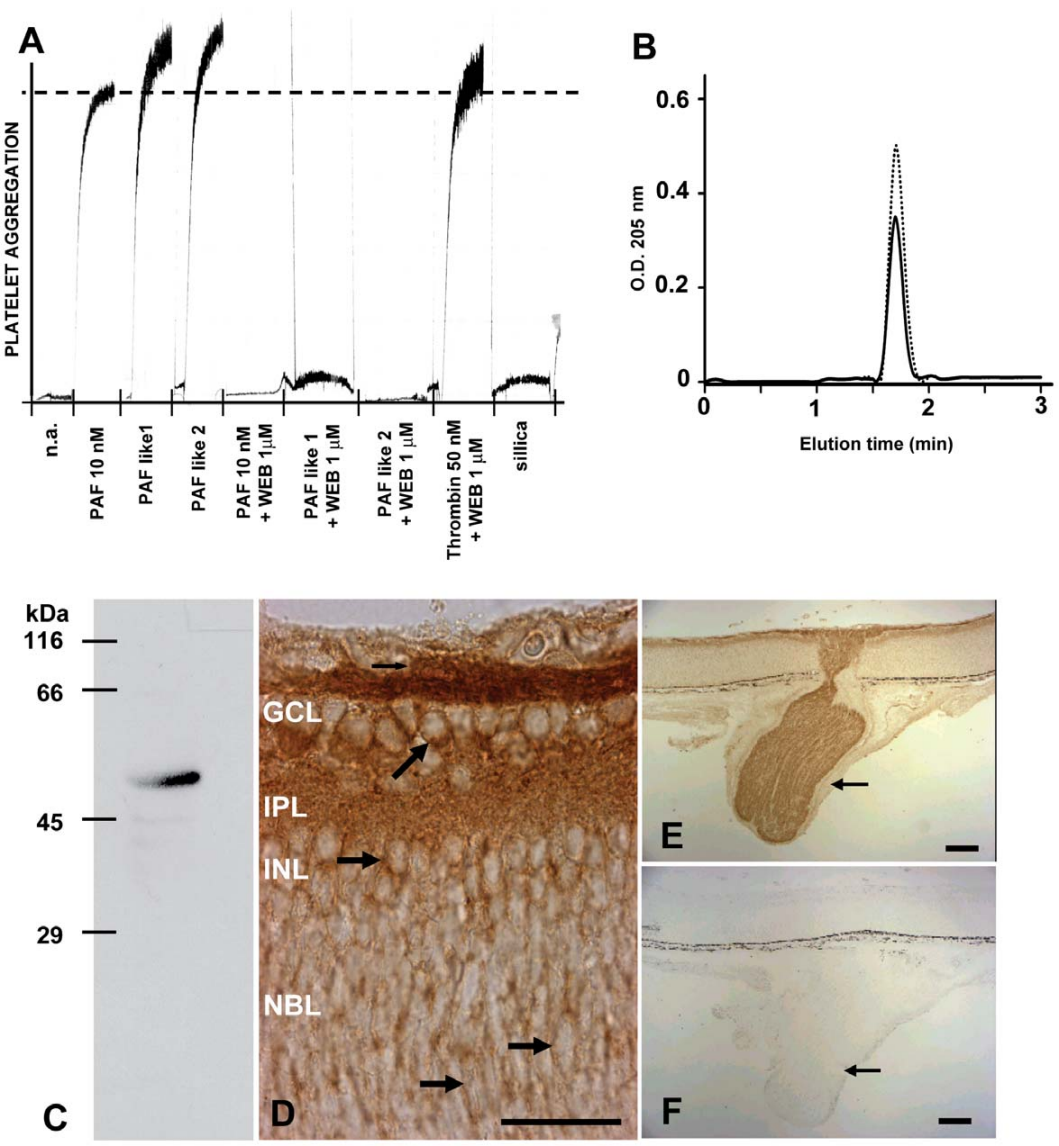
PAF and PAF receptor in the retina of developing rats. **A:** Washed rabbit platelets were treated with two independent samples of retina-derived PAF-*like*. Positive controls were commercial PAF (10 nM) and thrombin (50 nM), WEB2086 was used at 1 µM, and silica scraped from remote areas of the TLC plate was used as negative control. Note that the PAF receptor antagonist blocked platelet aggregation induced by retina-derived PAF-*like*, but not by thrombin; **B:** PAF-*like* subject to reverse phase HPLC eluted as a single peak (continuous line), coincident with pure commercial PAF (dotted line); **C:** Western blot of PAFR among a total protein extract from P2 rat retina shows a single band. **D–F:** Immunohistochemical detection of PAF receptor in transverse sections of newborn rat eyes. A negative control without primary antibody is shown in F. Arrows in D show labeling consistent with a membrane receptor in various retinal layers; Arrows in E, F show optic nerve axons. GCL  =  ganglion cell layer; IPL  =  inner plexiform layer; INL  =  inner nuclear layer; NBL  =  neuroblastic layer. Scale bars in D = 50 µm; E, F = 100 µm.

PAF-*like* from the neonatal rat retina induced platelet aggregation, similar to commercial PAF. The PAFR antagonist WEB 2086 prevented platelet aggregation induced by retinal PAF-*like*, but not aggregation induced by thrombin (Fig. 1A). HPLC showed a single peak of absorbance of PAF-*like* at the same elution time as commercial PAF (Fig. 1B), whereas the HPLC peak of phosphatidylethanolamine was distinct (Figure S3B). The combined biological and biochemical results strongly suggest that retinal PAF-*like* was either PAF itself or both a structurally- and functionally-related lipid.

To test which retinal cells produce PAF-*like*, separate bi-dimensional TLC preparations were made of: the posterior half of the eye, containing the sclera, choroid and immature pigment epithelium; the vascular network apposed to the vitreal margin of the retina, which contains vascular endothelium, pericytes and scattered macrophages [45]; purified Muller glial cell cultures, and compared with extracts from neural retina. Among the extracts examined with the current technique, only those from Muller cells showed a distinctive spot at the expected PAF-*like* location, suggesting that this major glial cell type of the developing retina produces high amounts of PAF. The Muller cell cultures apparently produced even higher amounts of PAF than macrophages (Figure S4).

A single 55 kDa band, corresponding to the molecular mass of PAFR was detected by Western Blots of protein extracts from neonatal rat retina (Fig. 1C). In immunolabeled sections from the retina *in situ*, PAFR was found in all retinal layers, including the neuroblastic layer (Fig. 1D), and the pattern of labeling suggest that most, if not all, retinal cells at this stage contain PAFR. In contrast with the labeling consistent with the membrane form of PAFR (Fig. 1D, arrowheads), we found no evidence of an additional, previously described nuclear form of the PAF receptor [46]. Relevant to the aims of the present study, in single cells dissociated from the neonatal retina, immunolabeling for PAFR was found both in non-proliferating cells, and in those expressing the cell cycle antigen Ki67 (Figure S5), thus confirming that retinal progenitors contain the PAF receptor.

### PAF blocks interkinetic nuclear migration

**Figure 2:**
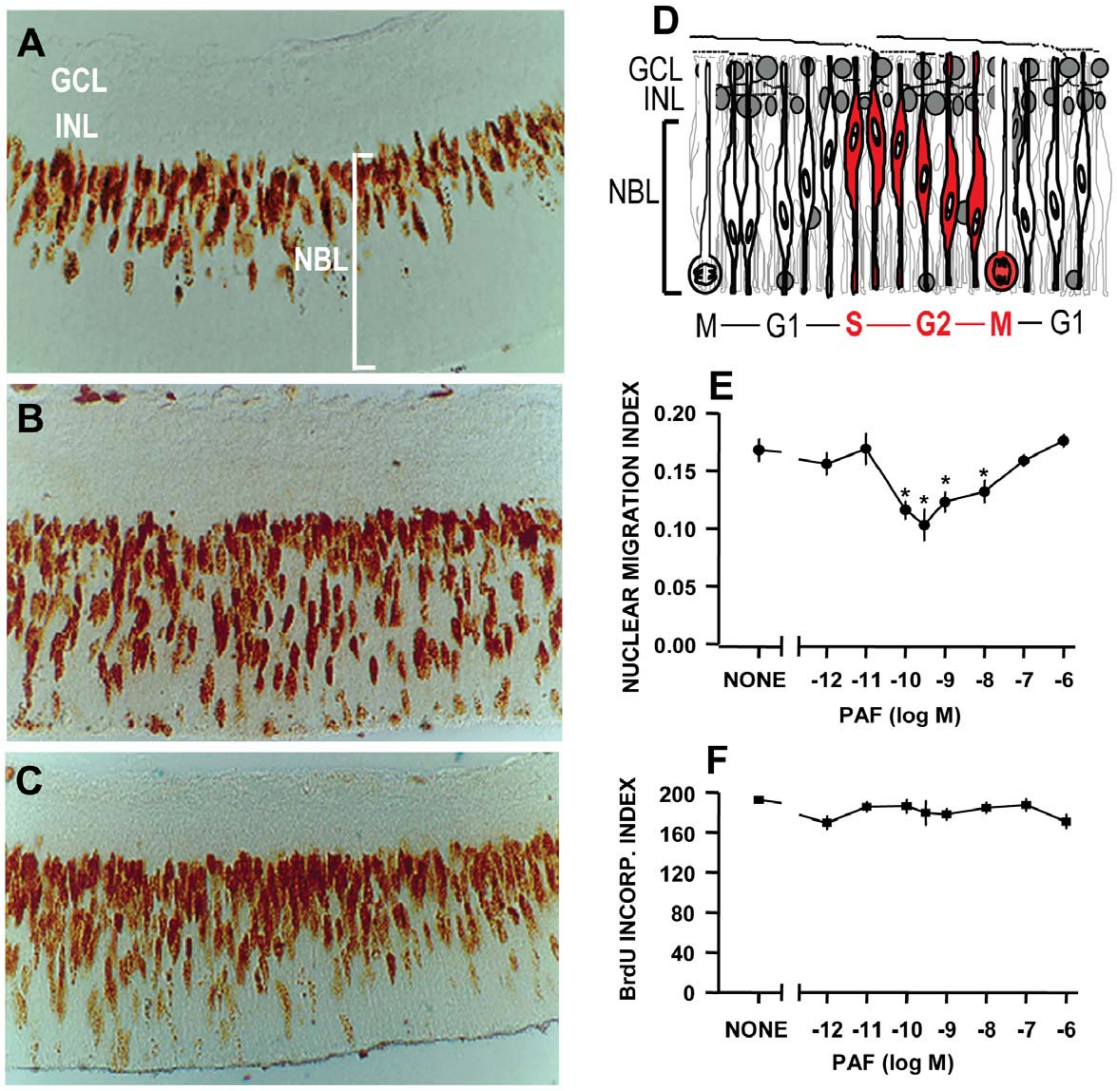
PAF blocks interkinetic nuclear migration. **A–C:** Sections of retinal explants immediately after dissection (A), at 3 h of incubation either in control medium (B), or with 0.3 nM PAF (C); **D:** A schematic diagram of interkinetic nuclear migration in the developing retina. Red elements mark the period of the cell cycle examined in the current study. **E:** Nuclear migration index in explants treated for 3 h with PAF. Notice the partial blockade of interkinetic nuclear migration in the range 10^−10^-10^−8^M. *  =  p<0.01 vs. control; **F:** BrdU incorporation index, showing a constant number of labeled nuclei across the NBL, irrespective of treatment with PAF; Data are means ± S.E.M., n = 3 in duplicate. *  =  p<0.01; **  =  p<0.001. Abbreviations as in Fig. 1.

To examine the effects of PAF upon IKNM (Fig. 2D), proliferating cells in S phase were pulse-labeled with BrdU *in vivo*. Starting at 45–60 min after injection, retinal explants were treated with various concentrations of PAF for an additional 3 h, and a nuclear migration index was calculated as described in Methods. PAF reduced the fraction of outward migrating nuclei with the maximum effect of approximately 50% at 0.3 nM (Fig. 2A–C, E). The hydrolysis-resistant PAF analog C-PAF also reduced the nuclear migration index by a maximum of approximately 45% (Table S1).

PAF did not affect the total number of BrdU-labeled nuclei within the counting fields (Fig. 2F), and, despite our searching for apoptotic profiles in every section examined throughout the study, we detected only occasional apoptotic profiles labeled with BrdU in either control or experimental explants (data not shown), thus ruling out that PAF induced programmed cell death in these experiments.

### Effect of PAF is receptor-dependent and involves multiple signal transduction pathways

**Figure 3:**
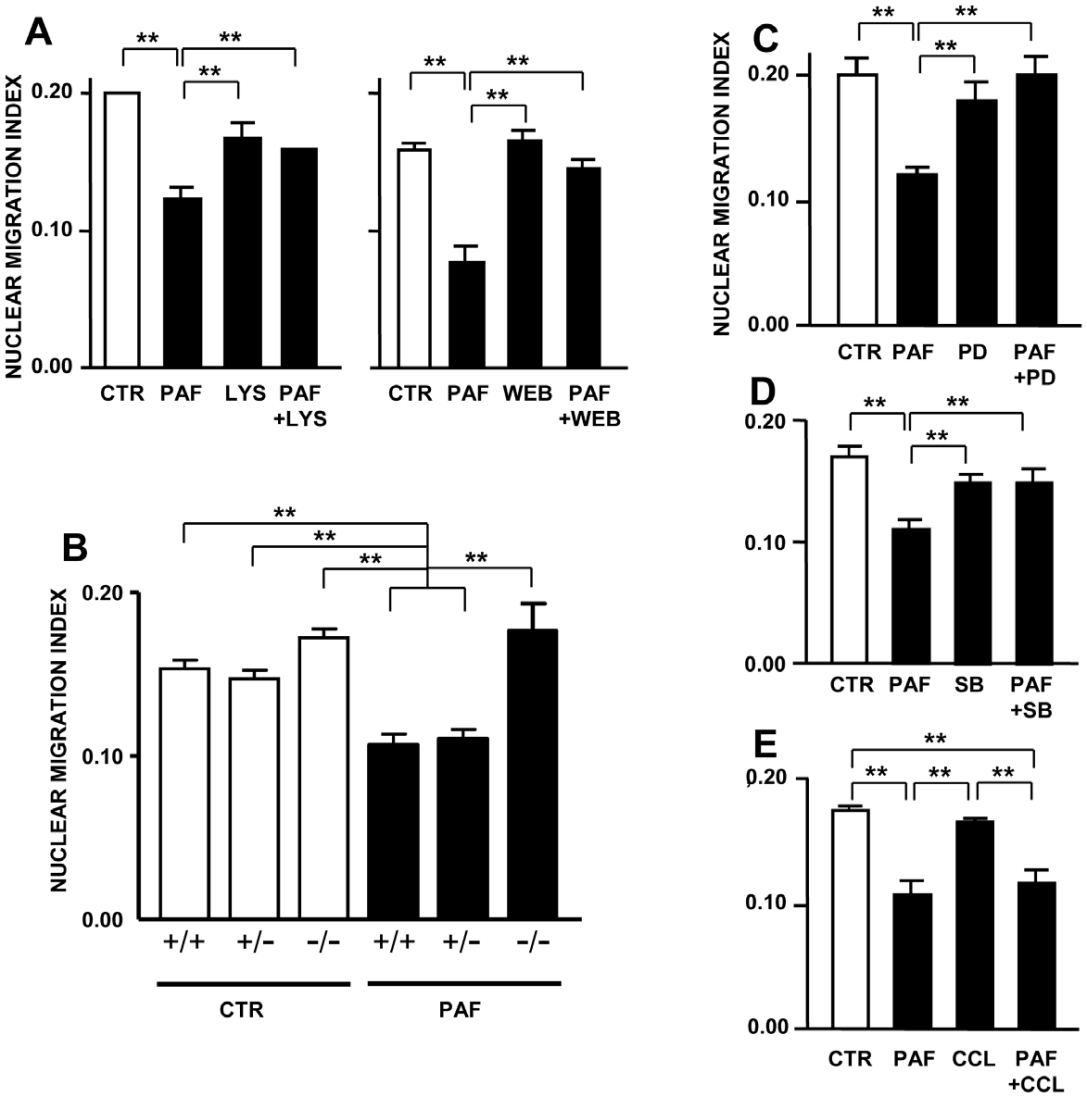
PAF-induced blockade of nuclear migration is receptor-mediated and modulated by several signal transduction pathways. P2 retinal explants, pre-labeled with BrdU, were treated with 0.3 nM PAF for 3 h in the presence of various PAF receptor (PAFR) antagonists or signaling inhibitors. **A:** Both a PAFR-ligand inactive PAF metabolite (Lyso-PAF 10 nM, left) and a PAFR antagonist (WEB2086 10 nM, right) prevented the effect of PAF; **B:** Deletion of PAFR abrogates the effect of PAF upon interkinetic nuclear migration; **C–E:** Antagonists of both Erk (C) and p38 (D) MAP kinases prevented the effect of PAF, whereas an inhibitor of protein kinase C (E) had no effect. Data are means ± S.E.M., n = 4 in duplicate. **  =  p<0.001.

Both the PAFR antagonist WEB2086 (10 nM) and the inactive receptor-binding metabolite lyso-PAF (1 nM-1 µM) (Fig. 3A) prevented the effects of PAF. Accordingly, no effects of the lipid mediator were detected upon IKNM in the retina of mice deficient for PAFR, whereas both wild-type and heterozygous mice were sensitive to blockade of IKNM by PAF (Fig. 3B).

Pre-treatment of retinal explants with either the p42/44 MAP kinase (Erk) inhibitor PD 98059 (20 µM), or with the p38 MAP kinase inhibitor SB 239063 (20 µM) prevented the effect of PAF (Fig. 3C,D). Both the PI3-kinase inhibitor LY 294002 (20 µM), and the adenylyl cyclase activator forskolin (10 µM), which stimulates the production of cAMP, also appeared to have similar, albeit not statistically significant effects (Table S1). In contrast, the PKC inhibitor chelerythrine chloride (1 µM) had no effect (Fig. 3E). Thus, several signaling pathways downstream of the plasma membrane PAFR are involved in the effect of PAF upon IKNM.

### Nuclei of proliferating cells at the end of the S-phase escape PAF-induced blockade

**Figure 4:**
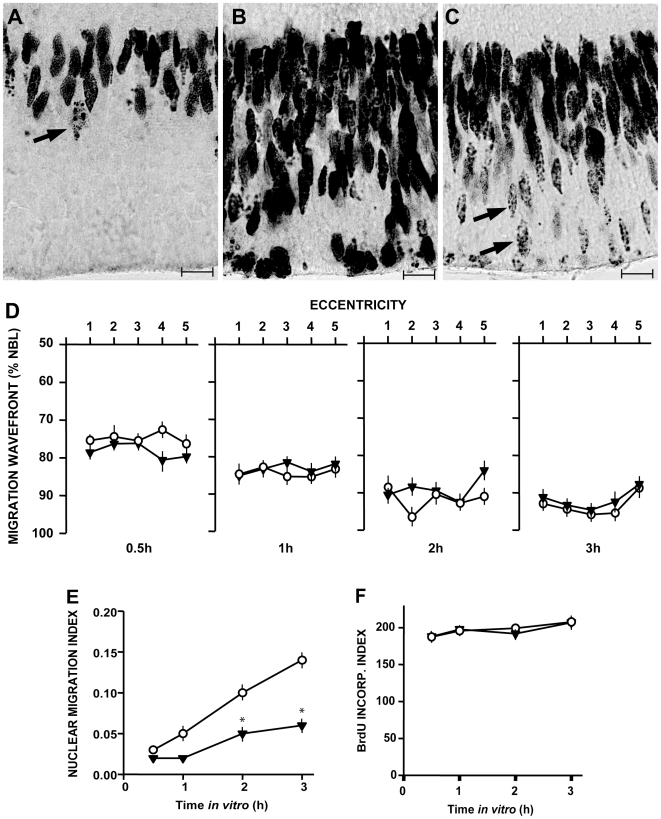
Nuclei of proliferating cells at the end of the S-phase escape PAF-induced blockade, and PAF does not interfere with nuclear movement proper. **A–C:** High magnification of immunolabeled sections of retinal explants immediately after dissection (A), at 3 h of incubation either in control medium (B), or with 1 nM PAF (C). Arrows highlight the mottled, predominantly peripheral pattern of BrdU incorporation in nuclei departing from the S-phase stratum (A), and in the reduced number of nuclei that reach the outer portions of the neuroblastic layer in PAF-treated explant (C), when compared with control (B). This labeling pattern is typical of nucleotide incorporation at the end of S-phase. Scale bar  = 10 µm. **D–F:** Radially cut P2 retinal explants were pulse-labeled with BrdU *in vitro* and treated with 0.3 nM PAF for 0.5, 1, 2 or 3 hours. Then, transverse sections immunolabeled for BrdU were used to map the location of the furthest migrating nuclei in 5 locations along the centro-peripheral extent of the retina (central = 1; peripheral = 5). The mean average distances (wavefronts) travelled by the furthest migrating nuclei are shown in **D**, as percentage of the basal-to-apical extent of the neuroblastic layer, as a function of retinal eccentricity from the optic disk. Open circles  =  control, filled triangles  =  PAF-treated. Note the coincident migration wavefronts, despite the distinct nuclear migration indexes (**E**, *  =  p<0.01 vs. control). Constant BrdU labeling index (**F**) is a control.

Close examination of the reduced fraction of BrdU-labeled nuclei that reached the outer third of the NBL after treatment with PAF, showed a heterochromatic pattern of nucleotide incorporation (Fig. 4). Labeling was mottled and often located at the nuclear periphery (Fig. 4C), quite distinct from the more homogeneous labeling of a large proportion of the nuclei that reached the apical border of the NBL in control explants (Fig. 4B). Labeling was also mottled in a few nuclear profiles found slightly displaced from the compact group of BrdU-labeled nuclei soon after injection of the nucleotide (Fig. 4A). This heterochromatic pattern of BrdU labeling is consistent with its incorporation at the end of S phase [47], followed by almost immediate outward migration. The data, therefore, indicate that in our experimental condition, only nuclei pre-labeled at the latest stages of the S phase escaped the action of PAF.

### PAF does not interfere with the velocity of nuclear movement proper

We then plotted the progressive displacement of a wavefront of migrating nuclei at various time points after pulse-labeling with BrdU *in vitro*. This allowed an estimate of the average speed of displacement of nuclei along the basal to apical extent of the NBL. The procedure was designed so as to avoid bias imposed by the centro-peripheral gradient of retinal differentiation, but we found no systematic differences along the central-peripheral axis, which corroborates the other data obtained from randomly distributed explants.

As expected, the longer the exposure to PAF, the greater was the difference between the nuclear migration indexes of either experimental or control explants (Fig. 4E). Nonetheless, the respective migration wavefronts were similar at all time points (Fig. 4D). Thus, once nuclei managed to exit the S phase stratum, they moved towards the apical border of the NBL with normal velocity, irrespective of treatment with PAF. Again, the BrdU incorporation index showed no difference between the two groups at any time point (Fig. 4F). These data show that the blockade of nuclear migration shown in Fig. 2 does not amount to an inability of retinal progenitor nuclei to move.

### Treatment with PAF does not block DNA synthesis nor causes DNA damage

**Figure 5:**
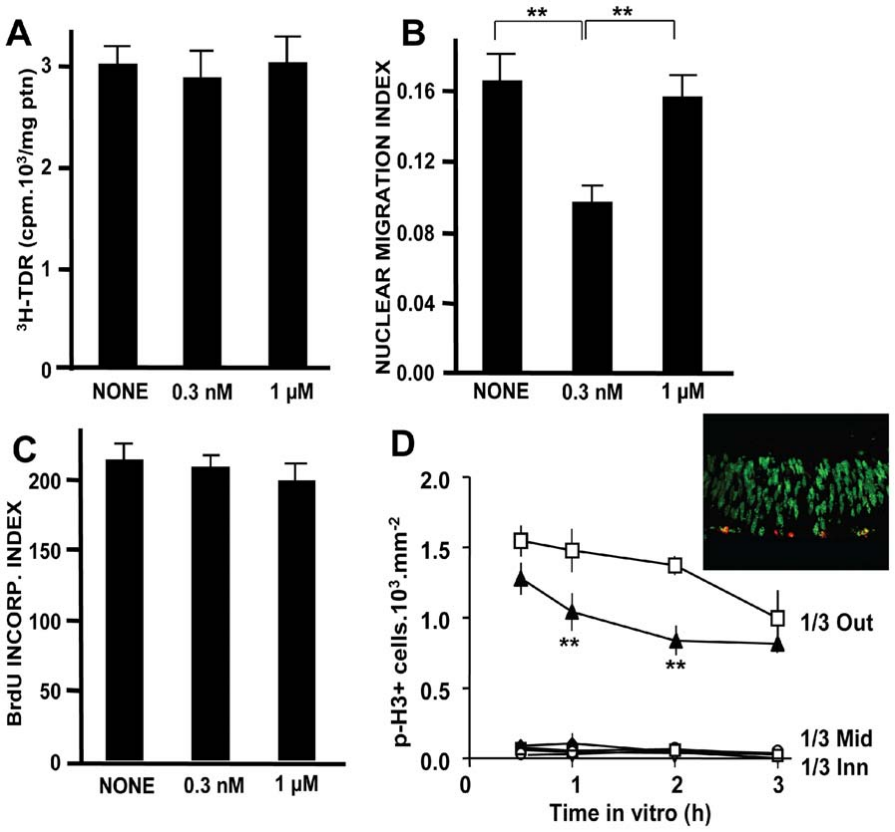
PAF induces a post-replication cell cycle arrest. **A–C:** Treatment with PAF does not block DNA replication. Tritiated thymidine was equally incorporated in BrdU-labeled control and PAF-treated explants (A), irrespective of blockade of interkinetic nuclear migration (B and C are controls from the same batch of explants used for the ^3^H-TDR measurements); **  =  p<0.001. **D:** Arrested nuclei do not progress along the cell cycle. Explants from the retina of animals pre-injected with BrdU were maintained either in control medium or treated with 0.3 nM PAF for various intervals, and retinal sections were stained with antibodies to BrdU (green in the inset) and to phospho-histone H3 (red). Despite the blockade of nuclear migration, no immunolabeling was detected among nuclei arrested within the inner 2/3 of the neuroblastic layer (inset), and all pH3-labeled nuclei were located within the outer (apical) 1/3, albeit with a distinctive reduction in PAF-treated explants. Data are means ± S.E.M., n = 4 in duplicate; **  =  p<0.001 vs. control at same time *in vitro*.

Since our initial hypothesis, that PAF would affect the movement of nuclei down the path of IKNM, does not fit the results above, we turned our attention to cell cycle checkpoints, the induction of which might affect the associated IKNM. We first tested whether PAF activated an intra-S checkpoint, which is typically induced by disruption of DNA replication, for example, by DNA damage [48]. The incorporation of nucleotides was similar to untreated explants following either the most effective concentration of 0.3 nM PAF or of 1 µM PAF, which does not block nuclear migration (Fig. 5A–C), and irrespective of whether tritiated thymidine was added at either the beginning or only during the last 1 hour of PAF treatment.

**Figure 6:**
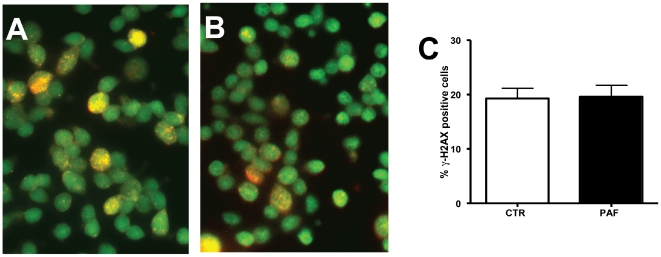
Lack of significant DNA damage induced by PAF. **A–B:** Photomicrographs of the nuclei of cells dissociated from retinal explants either untreated (A) or incubated with 0.3 nM PAF (B), double-labeled with the DNA marker SYTOX green, and with a monoclonal antibody to p-H2AX developed with a red fluorochrome-labeled secondary antibody. **C:** Frequency of cells showing foci of p-H2AX in cells from either untreated or PAF-treated retinal explants. Data are means ± S.E.M., pooled from 4 independent experiments.

Furthermore, there were no significant differences between the 2 groups in the percentages of cells containing clusters of γ-H2AX foci indicative of DNA damage [49] (Fig. 6). Thus, PAF did not induce DNA damage nor prevented DNA synthesis and, therefore, the blockade of IKNM does not correspond to the induction of a typical intra-S checkpoint.

### Migration-arrested nuclei do not progress to G2/M transition

The 3-hour incubation would have allowed enough time for nuclei to pass through G2 and enter mitosis [36]. We, thus, immunostained retinal sections for phosphorylated Histone H3, which labels retinal nuclei at late G2- and M-phases [38], and scored labeled nuclei along the IKNM path. No ectopic p-H3 labeling was detected in either control or PAF-treated retinae (Fig. 5D and inset). Interestingly, the number of p-H3 labeled nuclei was significantly reduced within the outer third of the NBL, suggesting that the impaired progression of proliferating cells along G2, induced by PAF, was not accompanied by cell cycle arrest at either the G2/M transition or during M-phase. We also did not notice any signs of mitotic chromosome condensation in BrdU-labeled nuclei outside of the apical margin of the retina.

### PAF prevents the build-up of cyclin B1 in arrested cells

**Figure 7:**
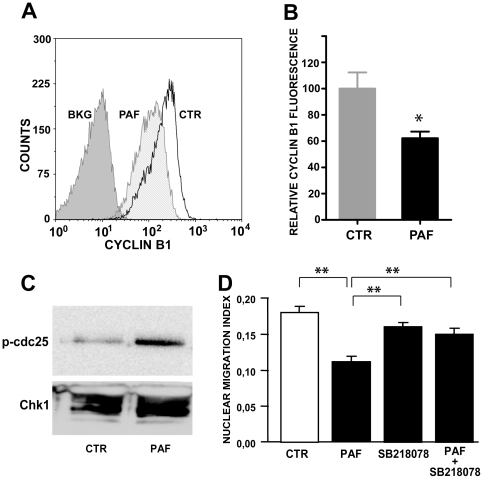
PAF blocks cyclin B1 increase during G2 phase, and PAF-induced cell cycle arrest depends on Chk1. **A, B:** Analysis of cyclin B1 content in BrdU-labeled cells. **A:** Cytofluorograms gated on BrdU-labeled cells dissociated from either control or PAF-treated retinal explants shows displacement of PAF-treated cells towards lower levels of cyclin B1; BKG  =  controls without primary cyclin B1 antibody; **B:** Mean and S.E.M. of the median cyclin B1 fluorescent intensity in BrdU-labeled cells, averaged among 4 independent experiments. Data were normalized to the respective controls in each experiment; *  =  p<0.01. **C:** Treatment of retinal explants with 0.3 nM PAF for 3 h induces an increase in the activity of Chk1, as shown by increased phosphorylation of a target cdc25C peptide (n = 2 with identical results) **D:** SB218078, a Chk1 inhibitor, blocks the effect of PAF (Data are means ± S.E.M., from n = 5 independent experiments in duplicate); **  =  p<0.001.

The data suggested an arrest at the S/G2 transition. We, therefore, tested whether PAF impaired the robust increase in the content of cyclin B1 expected during G2 phase. The results showed that BrdU-labeled cells were partially prevented from increasing their content of cyclin B1 (Fig. 7A, B). These data corroborate the hypothesis that PAF blocked the interkinetic nuclear migration by arresting the cell cycle at the S/G2 transition.

### PAF-induced arrest at the S/G2 transition depends on Chk1

The protein kinases ataxia teleangiectasia mutated (ATM), ATM-and-Rad3-related (ATR), and checkpoint kinases 1 and 2 (Chk1/Chk2) are major components of the mechanisms that oversee the control of DNA replication and genomic integrity [50]. The evidence of an arrest of retinal progenitor cells at the end of S-phase prompted a preliminary test of the involvement of those checkpoint components in the effect of PAF. Treatment with PAF induced activation of Chk1, as assayed *in vitro* by the phosphorylation of a peptide containing a known target serine (Fig. 7C). We, therefore, tested the effect of a Chk1 inhibitor upon nuclear migration. SB 218078 prevented the effect of PAF (Fig. 7D), indicating that Chk1 activity is required for the induction of the S/G2 cell cycle arrest. In contrast, caffeine, which inhibits the upstream ATM/ATR kinases, had no effect (Table S1).

## Discussion

The initial aim of this investigation was to test for an effect of PAF upon interkinetic nuclear migration in retinal progenitor cells. Indeed, PAF induced partial blockade of IKNM, via engagement of its cognate receptor, and mediated by activation of Chk1 through a signaling network that includes at least the Erk and p38 protein kinases. These data appeared consistent with the aforementioned modulation of cell and nuclear migration by both LIS1 and by PAF agonists and antagonists, as well as with structural evidence for overlapping domains of interaction of LIS1 with both the catalytic subunits of PAFAH and the molecular motor dynein [51].

Unexpectedly, however, mapping of the wavefront of migrating nuclei in the current experiments failed to show any evidence that the blockade of IKNM reflected impaired nuclear movement. This and the absence of ectopic mitoses also diverge from a previous study of the ventricular zone of the cerebral cortex [26], where it was suggested that the nuclei of LIS1 deficient cells moved slowly through G2 phase and therefore entered mitosis before reaching the apical end of the proliferating neuroblasts. The lack of an effect of PAF upon nuclear movement is in sharp contrast with the blockade of nuclear movement induced by inhibition of the CK2 enzyme, which was clearly shown with the same methods employed in the current study [13]. The present data, therefore, suggest that the effect of the lipid mediator upon IKNM in retinal progenitors is not related to the extensively studied roles of the regulatory subunit of PAFAH upon microtubule dynamics and molecular motor functions [52], [53], [54].

Indeed, the most significant result of our study was the unusual evidence for an S/G2 cell cycle arrest provided by a PAF-induced, Chk1-mediated mechanism. A DNA damage-induced intra-S checkpoint [48] was ruled out by both the lack of reduced nucleotide incorporation into DNA, as well as by the lack of evidence of PAF-induced DNA strand breaks. In turn, an arrest at the G2/M transition [55] was ruled out by the lack of H3 histone phosphorylation in the arrested nuclei, which would be expected to appear at late G2 just prior to chromosome condensation in mammalian cells [38], [56], as well as the lack of mitotic chromosome condensation in BrdU-labeled nuclei outside of the apical margin of the retina. The cell cycle arrest induced by PAF in retinal tissue is reminiscent of the induction by low-dose ultraviolet irradiation of a postreplication checkpoint in yeast [57].

The interpretation that PAF-treated retinal progenitor cells arrest at the S/G2 transition was further strengthened by the combination of the blockade of IKNM at the basal side of the proliferating cells with the abrogation of cyclin B1 build-up, which would be expected to occur along the S/G2 transition and during G2 [58], [59]. Indeed, IKNM facilitates the assignment of events to the S/G2 transition and early G2, as opposed to late G2 and the G2/M transition [55], [60], [61]. It would be interesting to investigate analogous events in non-neural tissue that also show IKNM, such as the pseudostratified epithelium of the developing liver bud [62].

PAF reportedly induces proliferation of various cells types ([29] for review), and the lipid activated both Erk and p38MAPK, and stimulated proliferation in an epidermal cell line transduced with PAFR [63]. In contrast, PAF inhibited proliferation and promoted differentiation of various colon carcinoma cell lines, in parallel with the activation of Erk, p38 and Jun N-terminal kinases [64].

The cell cycle inhibitory effect of PAF in the developing retina is in line with the data in colon carcinoma cells, and required the PAF receptor, as well as the activities of both the Erk and p38 MAP kinases. Differing cell cycle responses to PAF may be due to cell type-specific conditions, such as the availability of trans-interacting partners, as suggested for transduced epidermal cells [63]. Notwithstanding, it is not clear how PAF can either stimulate or inhibit the cell cycle while activating similar sets of signal transduction pathways.

In retinal tissue, PAF activated Chk1, and a selective inhibitor of Chk1 abrogated the lipid-induced blockade of IKNM. However, the ATM/ATR inhibitor caffeine had no detectable effect. These results suggest that the pathway of activation of Chk1 in the induction of the S/G2 arrest differs from those induced by irradiation [50]. Established models place p38 and Chk1/Chk2 at non-overlapping arms of the DNA damage response [65], and albeit p38 can phosphorylate Chk1, the former enzyme was ruled out as a significant player in a mitotic phosphorylation of the latter [66]. In turn, data were reported both that Erk is required for Chk1 phosphorylation and G2-M arrest induced by a microtubule disrupting agent in a colon cancer cell line [67], and that despite a requirement of the MEK/Erk pathway for an intrinsic cell cycle checkpoint that operates irrespective of DNA damage in *Drosophila*, the mechanism may function either together or in parallel with Chk1-dependent pathways [68]. We cannot, therefore, distinguish between either parallel or sequential involvement of Erk, p38 and Chk1 in the cell cycle arrest induced by PAF upon the retinal progenitor cells. In addition, since our measurement of phosphorylation of a substrate peptide revealed only the activity of Chk1, further investigation is needed to define among the various downstream targets of this enzyme [69], those relevant for signaling originated from PAF receptors.

The concentration dependency of PAF, corresponding to an inverted bell shaped curve (Fig. 2E), is typical of responses to this mediator [70], [71], [72], similar to other lipids [73], although it is unclear what are the relative contributions of mechanisms such as allosteric modulation [74], the presence of more than one variety of receptor with distinct affinities [75], or other unknown mechanisms [76], [77], [78]. Levels of PAF in 10 day-old postnatal rat retina *in vivo* have been estimated at about 15 pg/mg of protein, undergoing a 3–4 increase upon hyperoxia [79], whereas in 3-day posthatch chick retina, the best estimates amounted to roughly 1 ng/mg protein, undergoing a 5–6 fold increase following stimulation with acetylcholine, dopamine or a calcium ionofore [80]. It is, however, difficult to estimate the levels of extracellular PAF, because PAF-producing cells release only part of the lipid produced [80]. Nonetheless, the effective concentrations of PAF upon the retina in the present study are consistent with both the physiological concentration of PAF in other bodily fluids (within the 10^−12^-10^−8^ M range), and with the affinity of the PAF receptor for its ligand (K_d_ = 0.6 nM) [81], [82].

Our data suggest the operation in retinal neuroblasts of a novel, PAF-sensitive postreplication checkpoint that allows for cell cycle arrest at the completion of DNA synthesis prior to the departure of migrating nuclei at the outset of G2 phase. This concurs with the regulation of the neural cell cycle by molecules traditionally associated with immune responses [83], [84], [85], which may be released by glial cells during neural development and, possibly, also in the context of the proliferation of adult neural progenitors and stem cells. Indeed, our study showed that Muller cell cultures contain an unexpectedly large amount of PAF. The relevance of the latter data for the normal cell cycle is not yet clear, due to the difficulty in extrapolating the findings in Muller cell cultures to the developing retina *in situ*. Nonetheless, the Muller glia, which are abundant at postnatal day 2, together with other cell types, such as macrophages and microglia of the rat retina [45], [86], may provide PAF to receptor-bearing cells throughout the neuroblastic layer.

The production of PAF fluctuates during the cell cycle in yeast, with the lowest content in S phase, related to the availability of precursor and the activity of its synthesizing enzyme [87]. In turn, a specific population of neurons, the horizontal cells of the chick retina, lingers for approximately two days arrested at the G2 phase of the cell cycle following the last round of DNA replication, before they are released to progress through the M-phase of the neurogenic division [88]. Both these data are consistent with the suggested PAF-induced postreplication checkpoint. The functional consequences of this effect of PAF remain to be examined in detail in both the nervous system and other cell types.

Admittedly, the lack of an effect of the PAF antagonist upon basal IKNM, as well as the similar rates of IKNM in wild-type and PAF receptor-null mouse retinae, call into question physiological correlates of the PAF-induced blockade of IKNM. Nonetheless, given the variety of lipids active upon the retina [89], [90], [91], functions of PAF may be compensated by other lipid mediators in PAFR-null mice. Indeed, compensatory mechanisms have been shown in single gene knockout mice, whose lack of an expected phenotype apparently challenged otherwise demonstrated compelling evidence for functions of the cognate proteins (e.g. [92], [93]). Redundancy in the regulation of an event as crucial as IKNM for neural development would be expected, similar to what has been shown for various major components of cell cycle control [93]. Thus, further studies are warranted to effectively assess the role of PAF in normal retinal development. Alternatively, the lipid induced cell cycle arrest during retinal progenitor cell proliferation may be akin to the postulated immune responses to danger signals [94]. In this regard, possible links between the PAF-induced cell cycle arrest and oxidative stress associated with diseases such as retinopathy of prematurity, diabetic retinopathy and ischemic retinopathy [79], [95] deserve investigation.

## Supporting Information

Figure S1 **Analysis of interkinetic nuclear migration.** The top sequence illustrates the procedure for labeling proliferating cells with intraperitoneal injections of BrdU *in vivo*, retinal dissection and explantation, incubation, sectioning and immunohistochemistry. The lower panels illustrate the counting method. A counting field is delimited by the basal to apical extent of the neuroblastic layer. Counts are made of all labeled nuclei (nNBL) and, separately, of the nuclei located within the apical third of the counting field (nOUT1/3). The nuclear migration and BrdU incorporation indexes are explained in the frames. The lower right graph is a plot of the evolution of the nuclear migration index with time along the 3 hours of a typical experiment.
(TIF)

Figure S2 **Analysis of the wavefront of migrating nuclei.** In this particular experiment, the explants were prepared through radial cuts (red interrupted lines at the lower left), and BrdU was given after retinal explantation. Explants were collected at various intervals following the beginning of incubation with PAF, together with untreated controls. The photomicrograph shows one section immunolabeled for BrdU, and the counting procedure, as done on photomicrographs taken through a light microscope. At each of 5 evenly spaced locations in sections taken along the central (C) to peripheral (P) extent of the retina, a white line was drawn through the neuroblastic layer, and parallel black lines at 50 µm on each side delimited the scoring field. Within either side of the scoring field, the distance from the basal edge of the NBL to the furthest migrating nucleus was measured. The average of the 2 values was expressed as a percentage of the total length of the neuroblastic layer (white line) to provide one datum for that particular location. As described in methods, data were collected from the 4 time points in 2 independent duplicate experiments, each from 3–4 explants per data point, to provide a time course of advance of the furthest migrating nuclei, along the centro-peripheral axis of retinal eccentricity.
(TIF)

Figure S3 **Additional data on the identification of a PAF-like lipid in the developing retina.** **A:** Thin layer chromatography of commercial PAF, and of lipids of the neonatal retina, extracted in either ethanol (ETOH) or chloroform (CHCl_3_). Corresponding spots of commercial PAF and retinal PAF-like lipids are circled. **B:** Reverse phase HPLC of commercial PAF and of retina-extracted phosphatydilethanolamine (PE), used as controls of elution in HPLC experiments.
(TIF)

Figure S4 **Bi-dimensional Thin Layer Chromatograms of lipids extracted.** **A:** From the neural retina of postnatal (P) day 2 rat. **B:** From the vascular network apposed to the vitreal margin of the retina of P2 rats, which contains vascular endothelium, pericytes and scattered macrophages [45]. **C:** From the posterior half of the eye of P2 rats, containing the sclera, choroid and immature pigment epithelium. **D:** From the purified Muller glial cell cultures. Abbreviations: LPC  =  lysophosphatidylcholine; PC  =  phosphatidylcholine; PE  =  phosphatidylethanolamine; PI  =  phosphatidylinositol; PS  =  phosphatidylserine; PAF  =  platelet activating factor
(TIF)

Figure S5 **Neonatal rat retina was dissociated, and cells plated on poly-L-lysine coated coverslips were immunostained for Ki67 (green) and for PAF receptor (red), and counterstained with DAPI (blue).** The photomicrograph taken with epifluorescence in an Axiophot microscope shows several triple labeled profiles indicating the expression of PAF receptor in proliferating retinal progenitor cells.
(TIF)

Table S1 Effects of various treatments upon nuclear migration index
(DOC)
