# Supplementary figures and images for: Platelet Activating Factor Blocks Interkinetic Nuclear Migration in Retinal Progenitors through an Arrest of the Cell Cycle at the S/G2 Transition
Source: PLoS One. 2011 Jan 27;6(1):e16058. doi: 10.1371/journal.pone.0016058 (PMC3029264; doi:10.1371/journal.pone.0016058)

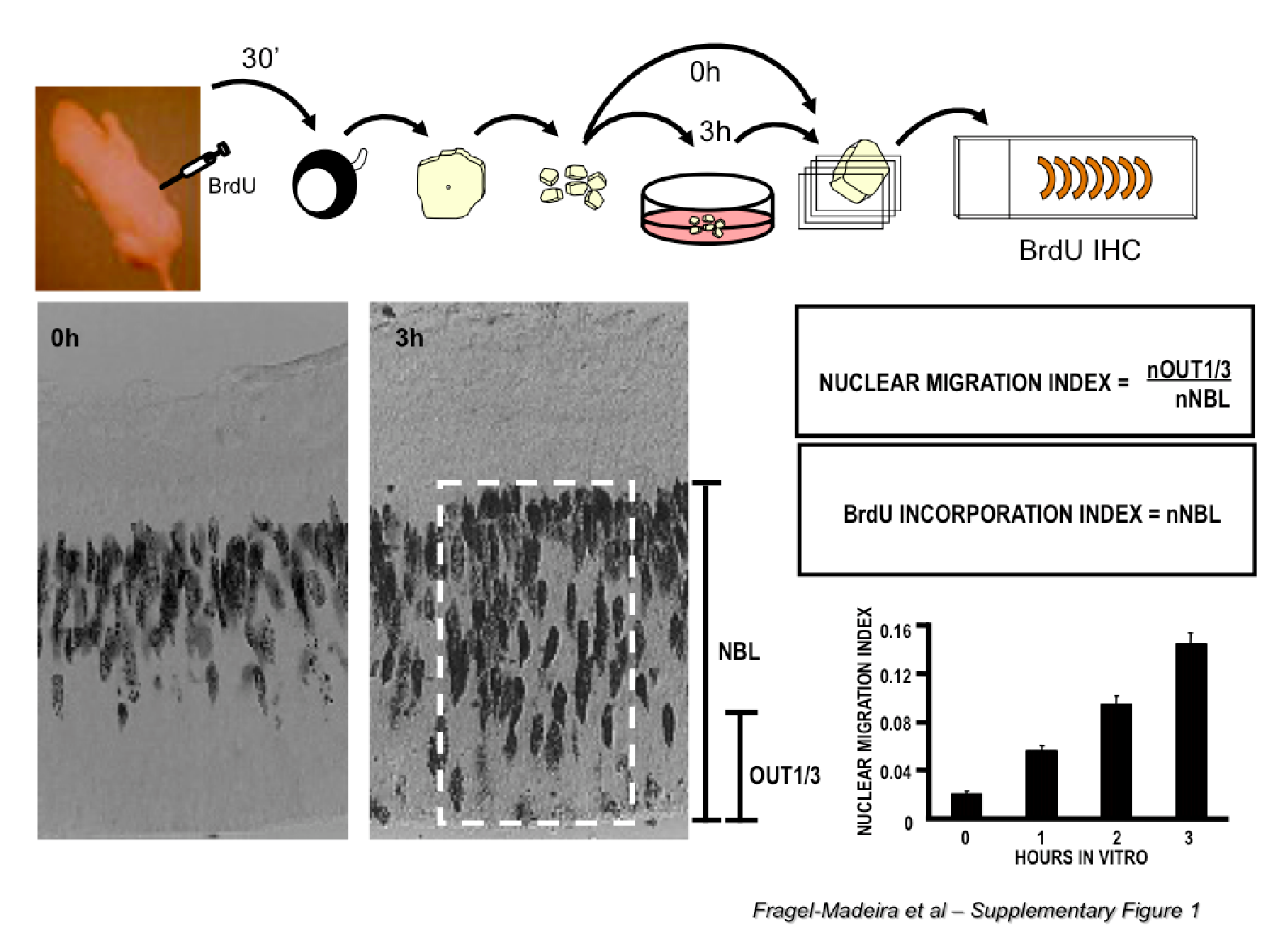

Supplement: Figure S1 — Analysis of interkinetic nuclear migration. The top sequence illustrates the procedure for labeling proliferating cells with intraperitoneal injections of BrdU in vivo, retinal dissection and explantation, incubation, sectioning and immunohistochemistry. The lower panels illustrate the counting method. A counting field is delimited by the basal to apical extent of the neuroblastic layer. Counts are made of all labeled nuclei (nNBL) and, separately, of the nuclei located within the apical third of the counting field (nOUT1/3). The nuclear migration and BrdU incorporation indexes are explained in the frames. The lower right graph is a plot of the evolution of the nuclear migration index with time along the 3 hours of a typical experiment. (TIF) [file pone.0016058.s001.tif]

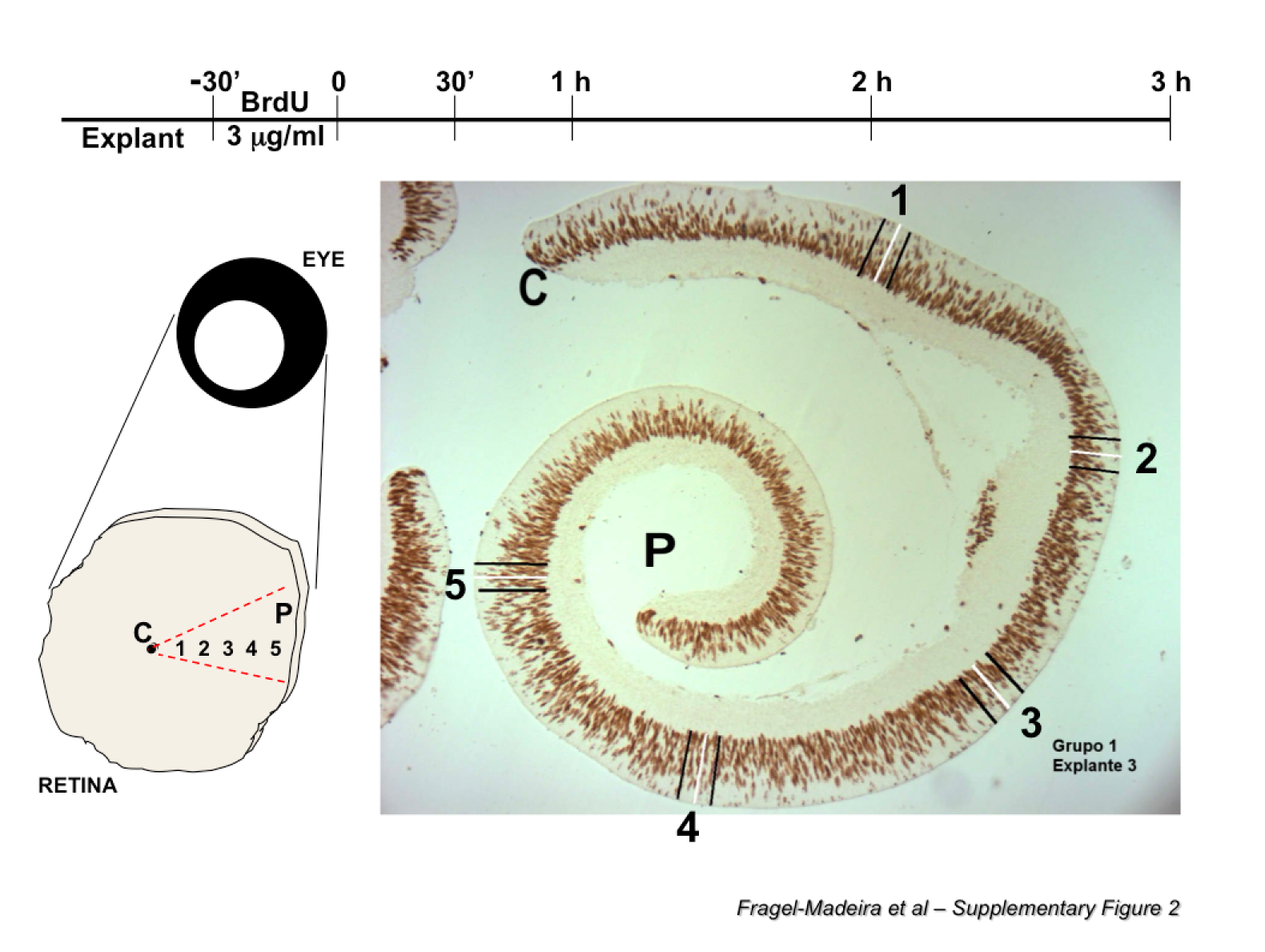

Supplement: Figure S2 — Analysis of the wavefront of migrating nuclei. In this particular experiment, the explants were prepared through radial cuts (red interrupted lines at the lower left), and BrdU was given after retinal explantation. Explants were collected at various intervals following the beginning of incubation with PAF, together with untreated controls. The photomicrograph shows one section immunolabeled for BrdU, and the counting procedure, as done on photomicrographs taken through a light microscope. At each of 5 evenly spaced locations in sections taken along the central (C) to peripheral (P) extent of the retina, a white line was drawn through the neuroblastic layer, and parallel black lines at 50 µm on each side delimited the scoring field. Within either side of the scoring field, the distance from the basal edge of the NBL to the furthest migrating nucleus was measured. The average of the 2 values was expressed as a percentage of the total length of the neuroblastic layer (white line) to provide one datum for that particular location. As described in methods, data were collected from the 4 time points in 2 independent duplicate experiments, each from 3–4 explants per data point, to provide a time course of advance of the furthest migrating nuclei, along the centro-peripheral axis of retinal eccentricity. (TIF) [file pone.0016058.s002.tif]

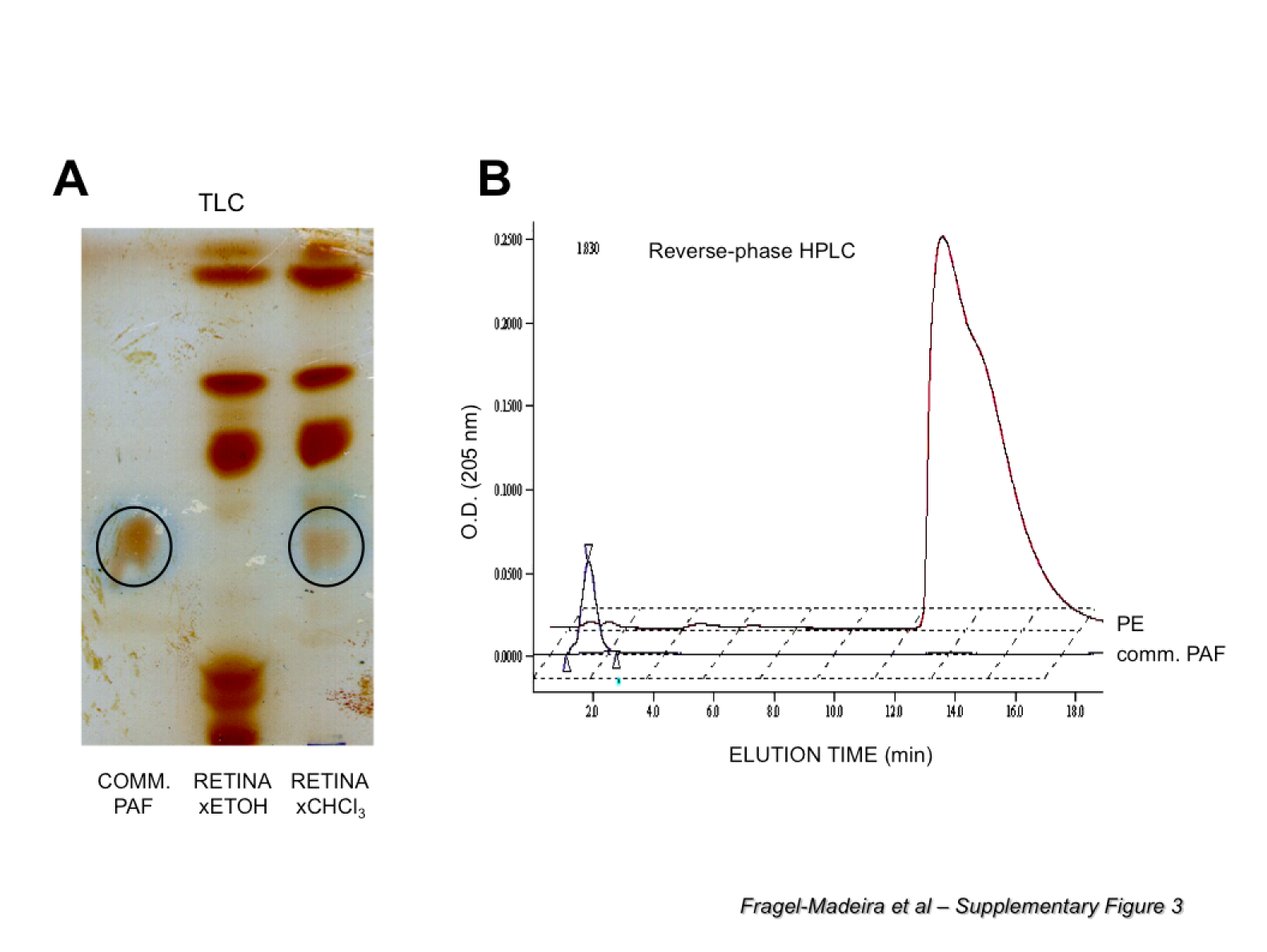

Supplement: Figure S3 — Additional data on the identification of a PAF-like lipid in the developing retina. A: Thin layer chromatography of commercial PAF, and of lipids of the neonatal retina, extracted in either ethanol (ETOH) or chloroform (CHCl3). Corresponding spots of commercial PAF and retinal PAF-like lipids are circled. B: Reverse phase HPLC of commercial PAF and of retina-extracted phosphatydilethanolamine (PE), used as controls of elution in HPLC experiments. (TIF) [file pone.0016058.s003.tif]

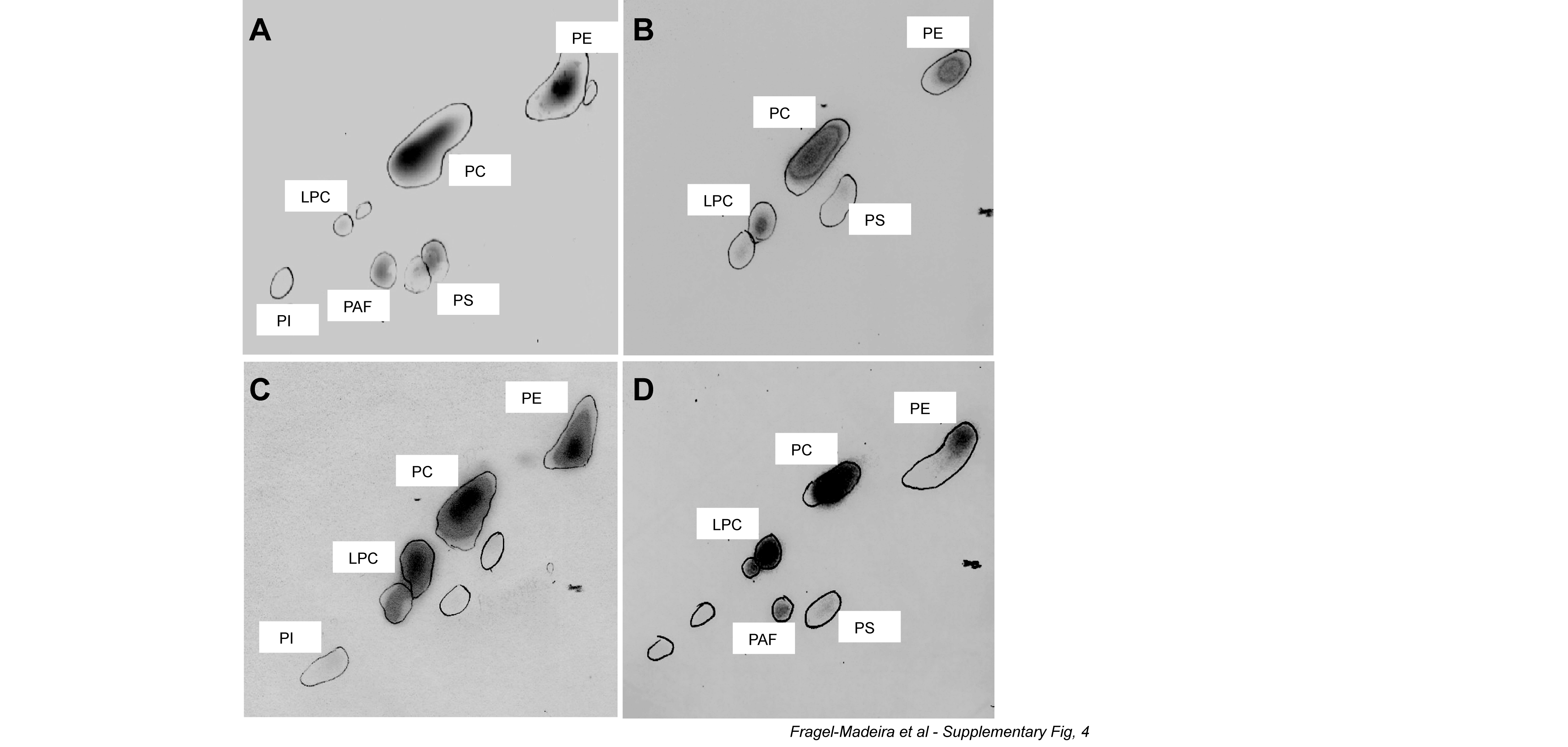

Supplement: Figure S4 — Bi-dimensional Thin Layer Chromatograms of lipids extracted. A: From the neural retina of postnatal (P) day 2 rat. B: From the vascular network apposed to the vitreal margin of the retina of P2 rats, which contains vascular endothelium, pericytes and scattered macrophages [45]. C: From the posterior half of the eye of P2 rats, containing the sclera, choroid and immature pigment epithelium. D: From the purified Muller glial cell cultures. Abbreviations: LPC = lysophosphatidylcholine; PC = phosphatidylcholine; PE = phosphatidylethanolamine; PI = phosphatidylinositol; PS = phosphatidylserine; PAF = platelet activating factor (TIF) [file pone.0016058.s004.tif]

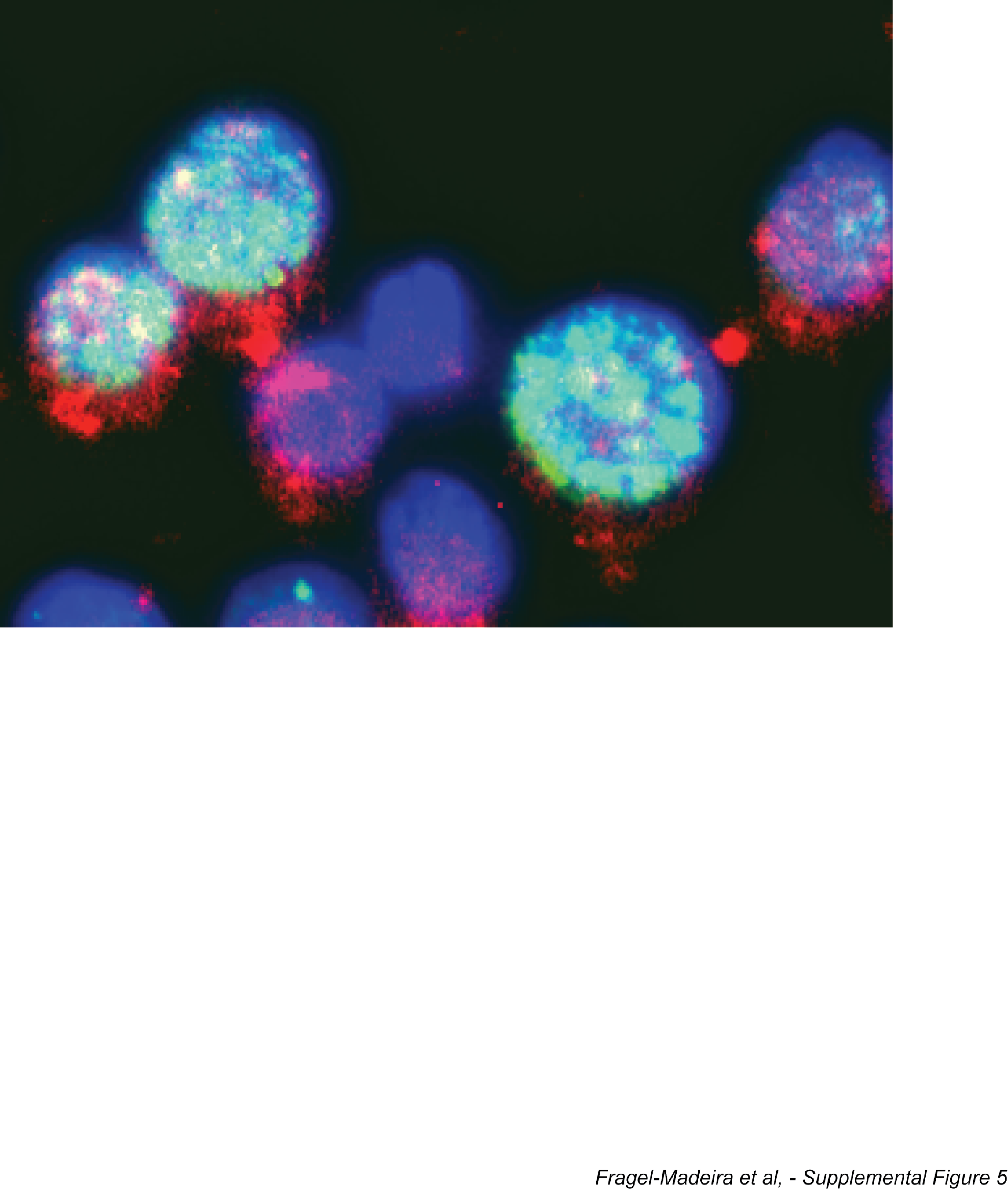

Supplement: Figure S5 — Neonatal rat retina was dissociated, and cells plated on poly-L-lysine coated coverslips were immunostained for Ki67 (green) and for PAF receptor (red), and counterstained with DAPI (blue). The photomicrograph taken with epifluorescence in an Axiophot microscope shows several triple labeled profiles indicating the expression of PAF receptor in proliferating retinal progenitor cells. (TIF) [file pone.0016058.s005.tif]
