# Supplementary material for: Platelet Activating Factor Blocks Interkinetic Nuclear Migration in Retinal Progenitors through an Arrest of the Cell Cycle at the S/G2 Transition
Source: PLoS One. 2011 Jan 27;6(1):e16058. doi: 10.1371/journal.pone.0016058 (PMC3029264; doi:10.1371/journal.pone.0016058)
Supplement: Table S1 — Effects of various treatments upon nuclear migration index (DOC) [file pone.0016058.s006.doc]

**Table S1: Effects of various treatments upon nuclear migration index**

| **TREATMENT** | **MEAN ± S.E.M. NUCLEAR MIGRATION INDEX (n)** |
| --- | --- |
|  |  |
| none | 0.16 ± 0.01 (3) |
| C-PAF 0.01 nM | 0.11 ± 0.01 (3) |
| C-PAF 0.1 nM | 0.12 ± 0.01 (3) |
| C-PAF 0.3 nM | 0.13 ± 0.01 (3) |
| C-PAF 1 nM | 0.10 ± 0.01 (3)* |
| C-PAF 10 nM | 0.12 ± 0.01 (3) |
| C-PAF 100 nM | 0.09 ± 0.01 (3)** |
| C-PAF 1 μM | 0.14 ± 0.01 (3) |
|  |  |
| none | 0.17 ± 0.02 (3) |
| PAF 0.3 nM | 0.11 ± 0.01 (3)*** |
| LY294002 20 μM | 0.18 ± 0.02 (3) |
| PAF 0.3 nM + LY294002 20 μM | 0.16 ± 0.02 (3) |
|  |  |
| none | 0.18 ± 0.01 (5) |
| PAF 0.3 nM | 0.11 ± 0.01 (5)*** |
| Forskolin 10 μM | 0.14 ± 0.01 (5) |
| PAF 0.3 nM + Forskolin 10 μM | 0.15 ± 0.01 (5) |
|  |  |
| none | 0.19 ± 0.01 (2) |
| PAF 0.3 nM | 0.13 ± 0.01 (2)** |
| PAF + caffeine 100 μM | 0.15 ± 0.01 (2)** |
| PAF + caffeine 300 μM | 0.13 ± 0.01 (2)** |
| PAF + caffeine 1 mM | 0.14 ± 0.01 (2)** |
| PAF + caffeine 3 mM | 0.15 ± 0.01 (2)** |

n = number of duplicate experiments; * p<0.05; ** p<0.01; *** p<0.001
